# The Effects of Microcrystalline Cellulose Addition on the Properties of Wood–PLA Filaments for 3D Printing

**DOI:** 10.3390/polym16060836

**Published:** 2024-03-18

**Authors:** Daša Krapež Tomec, Manfred Schöflinger, Jürgen Leßlhumer, Urška Gradišar Centa, Jure Žigon, Mirko Kariž

**Affiliations:** 1 Biotechnical Faculty, University of Ljubljana, Jamnikarjeva 101, 1000 Ljubljana, Slovenia; dasa.krapez.tomec@bf.uni-lj.si (D.K.T.); jure.zigon@bf.uni-lj.si (J.Ž.); 2 Kompetenzzentrum Holz GmbH, Science Park 2, Altenberger Straße 69, 4040 Linz, Austria; m.schoeflinger@wood-kplus.at (M.S.); j.lesslhumer@wood-kplus.at (J.L.); 3 Faculty of Mechanical Engineering, University of Ljubljana, Pot za Brdom 104, 1000 Ljubljana, Slovenia; urska.gradisarcenta@fs.uni-lj.si

**Keywords:** microcrystalline cellulose, thermally modified wood, wood–plastic filaments, rheological properties

## Abstract

This paper describes the use of microcrystalline cellulose (MCC) as an additive in wood-polylactic acid (PLA) filaments suitable for 3D printing. Filaments prepared with PLA, thermally modified (TM) wood, and three different MCC loadings (1, 3, and 5 wt%) by two-step melt blending in the extruder were characterized with respect to their rheological, thermal, and mechanical response. The analyses demonstrate that a low MCC content (1%) improves the mobility of the polymer chains and contributes to a higher elasticity of the matrix chain, a higher crystallinity, a lower glass transition temperature (by 1.66 °C), and a lower melting temperature (by 1.31 °C) and leads to a higher tensile strength (1.2%) and a higher modulus of elasticity (12.1%). Higher MCC loading hinders the mobility of the polymer matrix and leads to a rearrangement of the crystal lattice structure, resulting in a decrease in crystallinity. Scanning electron micrographs show that the cellulose is well distributed and dispersed in the PLA matrix, with some agglomeration occurring at higher MCC levels. The main objective of this study was to develop and evaluate a filament containing an optimal amount of MCC to improve compatibility between wood and PLA, optimize melt processability, and improve mechanical properties. It can be concluded that a 1% addition of MCC favorably changes the properties of the wood–PLA filaments, while a higher MCC content does not have this effect.

## 1. Introduction

The utilization of bio-derived materials in additive manufacturing is on the rise, driven by the ongoing need for innovation in sustainable materials and adherence to circular economy principles within the manufacturing industries [1]. One of the most widely used additive manufacturing (AM) processes, fused filament fabrication (FFF), has been used extensively in recent years to produce customized parts with complicated geometries, particularly from synthetic thermoplastics. Biodegradable polymers like polylactic acid (PLA) are increasingly being considered as alternatives to petroleum-based products. PLA, a thermoplastic semi-crystalline aliphatic polyester derived from renewable sources, is notable for its high strength, excellent optical transparency, and elevated elastic modulus compared to other synthetic polymers. Nonetheless, it also exhibits drawbacks such as brittleness, limited thermal stability, and a reduced capacity for crystallization [2]. With PLA as a pure material, three-dimensional (3D) structures can be produced that have relatively low strength and are easily deformed. To overcome these limitations, intensive work is being carried out on the development of new biodegradable PLA composite filaments for FFF that are reinforced with sustainable fillers (e.g., wood flour and microcrystalline cellulose (MCC)) [3]. In general, integrating cellulose fibrils into the PLA matrix yields composites characterized by biodegradability, enhanced crystallization, and improved mechanical and thermal properties when compared to the neat PLA matrix [4,5,6]. Cellulose offers several key advantages for 3D printing, primarily due to its high aspect ratio, widespread availability, ease of processing, sustainability (despite the use of intensive chemical processes in some cases), and potential for chemical functionalization. Cellulose stands as the most abundant natural polymer on Earth, characterized by its distinctive molecular structure and properties [7]. It is found in plant cell walls, but it can also be synthesized by some bacteria and animals. Cellulose has primarily been utilized in nanocomposites within hydrophilic environments, primarily because of the hydrophilic surface properties of cellulose fibrils. It is one of the most versatile biopolymers found in nature. Its use is now moving from sustainability-oriented development to technical solutions for a wide range of applications [8]. 

Cellulose is a macromolecule that generally has a combination of crystalline and amorphous regions [9]. In plants, cellulose serves as the main element for reinforcement and is widely distributed in all plant tissues. It mainly originated from wood—most commercial cellulose is obtained by pulping softwood or hardwood in which cellulose is abundant. Hardwoods and softwoods have a cellulose content of about 40–47% by weight in their composition [10]. The properties of fibers, as well as natural-fiber-reinforced composites, depend on their composition, microfibril angle, crystallinity, and internal structure [11]. Cellulose is composed of linear chains of β (1→4) linked D-glucopyranosyl units. These chains are hydrogen bonded to form microfibrils and microfibrillar aggregates, wherein highly ordered regions alternate with disordered regions [12]. 

While carbon fibers and glass fibers find applications in numerous composite applications, bio-based renewable alternatives are continuously being explored due to their sustainability and environmental impact. Cellulose fibers, available in various sizes and forms, can act as alternative reinforcement materials for AM polymers [13]. They are often used as reinforcing materials due to their high strength, high stiffness, and low density. Cellulose fibers extruded with thermoplastics are often used as filaments or in bio-ink formulations. Three-dimensionally printed cellulose-based composites have shown improved mechanical properties [14]. Micro- and nanocellulose have advantageous properties compared to commercially available fillers (e.g., talc and glass fibers), such as renewability, biodegradability, high surface area, high modulus, high strength, and low density. These renewable materials have some undesirable properties that encompass challenges such as constrained thermal stability, notably at typical melt processing temperatures around 200 °C, restricted compatibility with numerous thermoplastic matrices owing to their highly hydrophilic properties, inadequate dispersion characteristics within non-polar thermoplastic melts due to robust hydrogen bonding forces between the fibers and high moisture absorption by the fibers, adversely affecting the dimensional stability of composite materials [9].

MCC, obtained via acid hydrolysis that removes the amorphous regions, represents a promising cellulosic reinforcement for polymers. These are essentially crystalline cellulose extracted from high-quality pulp and are anticipated to degrade into cellulose whiskers following complete hydrolysis [15]. MCCs are porous particles, typically ranging from 10 to 50 μm in diameter, possessing high cellulose content and crystallinity. These particles consist of aggregate bundles of hydrogen-bonded cellulose microfibrils [16]. 

The hydroxyl (OH)-rich groups on the surface of cellulose fibers make them very hydrophilic, whereas most polymer resins are hydrophobic, resulting in poor dispersion and adhesion. Moreover, especially in the case of nanocellulose, these OH-rich groups lead to strong hydrogen bonding, which results in the agglomeration of these fibrils during the drying process [17]. Once agglomerated, it is almost impossible to redisperse these fibrils, even in water [13]. The primary challenge in developing composites lies in the inherent hydrophilicity of cellulose, which impacts its dispersibility and interfacial bonding with the matrix. In thermoplastic composites, acetylation of nanofibrillated cellulose (NFC) has emerged as a viable solution to enhance mechanical properties. PLA nanocomposites formulated with up to 5% acetylated NFC have demonstrated notable enhancements in Young’s modulus, tensile strength, and failure strain [18]. Formulations of PLA nanocomposites with up to 5% acetylated NFC showed a significant improvement in Young’s modulus, tensile strength, and failure strain [18].

A study by Tekinalp and co-authors [13] reported that the shear forces in the 3D printing process resulted in the alignment and stretching of nanofibrillated cellulose bundles in the printing direction, which further increased the stiffness and storage modulus. In other words, the printing process can achieve controlled directional stiffening of the manufactured parts.

The primary objective of incorporating natural fibers into polymer matrix composites is to lower the feedstock expenses while attaining a favorable stiffness-to-weight ratio, along with advantages such as recyclability, biodegradability, thermal insulation, and CO_2_ neutrality. These attributes distinguish them from conventional counterparts like glass fibers and carbon fibers [19]. Although the properties of natural-fiber-reinforced composites (NFRC) are in some cases inferior to those of composites with synthetic reinforcements, depending on the matrix and fiber combination, they are becoming increasingly important due to the advantages mentioned above. In addition, the production of natural fibers requires less energy (9.55 MJ/kg for flax) compared to synthetic fibers such as glass fibers (54.7 MJ/kg) [20].

In the context of sustainable production, composites consisting of polymer matrices and natural fibers are highly regarded due to their ability to offer desired properties and performance at a cost-effective rate. Despite their promising future potential, these composites face various material- and processing-related challenges that need to be addressed to ensure long-term stability and performance [21].

Although research in the field of bio-based polymers and their composites covers various materials, commercially available bio-based filaments are limited to PLA blends with wood-based materials. To make biodegradable cellulose-based composites competitive, research into industrially viable production techniques is important. In general, reinforced filaments are produced either by melt compounding in the extrusion process or by mixing fibers and polymers in the solvent-casting process with subsequent melt compounding. In most studies, nanocellulose was used as reinforcing material, with the content varying between 1 and 40%. Improvements of up to 84% in tensile strength [22] and 190% in stiffness were reported [8]. In some cases, the addition of cellulose had no positive effect on the mechanical properties. However, other improvements, such as thermomechanical stability and the rate of biodegradability, were observed [23]. A study by Bhasney and co-authors [24] came to the conclusion that the properties of PLA change significantly when MCC fibers are blended. While the complex viscosity of PLA and its biocomposites showed shear-thinning behavior in a frequency range of 0.1–100 rad/s, the storage modulus of PLA increased from low to high frequency. The water contact angle of the biocomposites decreased to about 62° after the addition of MCC fibers. Incarnato and co-authors [25] report that the rheological results demonstrated the addition of 12 wt% MCC does not change the rheological behavior of the PLA, while the addition of 18 wt% MCC significantly changes the trend and raises the viscosity of the PLA. Furthermore, the addition of MCC leads to a linear increase of the storage modulus at 35 °C with the filler loading, up to 47% for the sample PLA 18% MCC, while it has no effect on the phase transition temperatures of PLA. In a study by Haafiz [26] and co-authors, the addition of MCC resulted in a roughly 30% increase in the modulus of elasticity but a decrease in the composites’ tensile strength and elongation at break. According to SEM analysis of the composites’ fracture surface, the MCC continued to exist as crystalline cellulose aggregates. The improved compatibility between the matrix and the filler generally leads to better dispersion of the filler in the polymer matrix. This was one of the reasons why we tried to incorporate MCC into the wood–PLA filament. The aim of this study was to improve the properties of PLA by using MCC as reinforcement together with TM wood. The composites were produced by a two-step twin screw extrusion. The filaments and 3D-printed parts made from these materials were characterized to determine their rheological, thermal, and mechanical properties in order to investigate the effects of different contents of MCC on the viscoelastic response, the characteristic phase transition temperatures, and the strength of the 3D-printed parts.

Several studies have shown that the addition of nanocellulose (NC) has a positive effect on the mechanical, thermal, and rheological properties of materials [22,23,27]. The high production costs and the tendency to agglomeration hinder its widespread use. For this reason, MCC is currently present in many applications as commercially available micro-sized cellulose material [28]. The goal of this work was to produce fully biodegradable biocomposites for 3D printing using MCC as a reinforcing material. Our hypothesis was that sustainable microcrystalline cellulose improves the rheology and thermal stability of the filament and 3D-printed parts. Biodegradable composites based on thermally modified wood and PLA were prepared with MCC as reinforcing phase by two-step extrusion, followed by injection molding (for rheology) or 3D printing (for tensile, flexural strength, and scanning electron microscopy). The main objective of this study was to develop and characterize a wood–PLA filament with an ideal content of MCC to optimize its properties for application in 3D printing. As previously studied, the overall mechanical properties of polymer blends and composites depend on several factors, such as the aspect ratio of the additives, the crystallinity of the polymer matrix, the optimal number of additives, and the interfacial adhesion between the polymers and fillers [29]. Based on previous research [30], TM wood was used to ensure better compatibility with PLA. We have used MCC to reduce the production costs in the case of commercial filaments. To our knowledge, no study on the addition of MCC in TM wood–plastic filaments has been conducted to date.

## 2. Materials and Methods

PLA-type PLA Ingeo™ 2003D (NatureWorks, Blair, NE, USA) in granular form was used as the base material. The recommended melt temperature of the material is 210 °C, tensile strength at break 53 MPa (ASTM D882 [31]), specific gravity 1.24 g/cm^3^ (ASTM D792 [32]), melt flow index 6 g/10 min at 210 °C (ASTM D1238 [33]), and the molecular weight 200,000 g/mol.

Wood flour was prepared from beech (*Fagus sylvatica*) wood, thermally modified at 200 °C. To improve the interfacial adhesion between the PLA polymer and the wood, thermal modification was performed to ensure a lower hygroscopicity of the wood. 

Ultrafine microcrystalline cellulose powder Arbocel UFC 100 was kindly provided by J. Rettenmaier & Sohne (Rosenberg, Germany). UFC 100 has an average particle size (d 50) of 6 μm to 12 μm and a bulk density between 150 and 220 g/L.

### 2.1. Materials and Preparation

#### 2.1.1. Wood Particles Preparation

Kiln-dried beech boards were collected and cut into smaller lamellas 900 mm × 200 mm × 25 mm. They were dried to absolute dryness at (103 ± 2) °C for 24 h and weighed to later calculate the mass loss during thermal modification. The thermal modification was performed in a vacuum modification chamber (Kambič d.o.o., Semič, Slovenia) at 200 °C using the Silvapro method under semi-anoxic conditions [34]. The process of thermal modification was divided into the following three phases: (1) temperature increase (heating) to 200 °C, (2) modification process (constant temperature for 3 h), and (3) temperature decrease (cooling).

After thermal modification, the wood boards were weighed again to determine mass loss during modification and cut into small cubes of 20 mm × 20 mm × 20 mm. They were ground in a laboratory cutting mill SM 2000 (Retsch, Haan, Germany) first with a 1 mm sieve and in a second step with a 0.25 mm sieve. The obtained wood particles were then sieved through a 237 μm sieve and stored in airtight containers for further processing.

#### 2.1.2. Compounding

Before compounding, wood flour was dried at 103 °C for 24 h, and MCC and PLA granules were dried at 60 °C for 12 h [35].

The filament was produced in a two-step process to ensure better homogeneity. In the first step, the MCC was mixed with TM wood and a polymer matrix to form pellets. 

**Figure 1:**
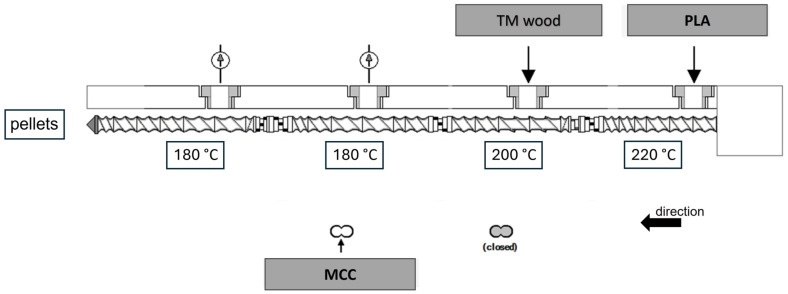
Compounding line set-up.

The components were compounded using a laboratory-scale twin-screw extruder (Brabender, Duisburg, Germany) with a screw diameter of 20 mm and a length of 40D. The compounding process (Figure 1) was performed in the laboratory; gravimetric feeders were used. Extruding temperatures were 220– 200– 180– 180 °C, respectively, from hopper to die. The screw speed was 375 rpm.

For pelletizing, the EUP50 underwater pelletizing system (Econ, Weisskirchen, Austria) was used. The pellets were dried at 80 °C for 8 h before further processing.

#### 2.1.3. Filament Extrusion

A KDSE Mark II extruder (Brabender, Duisburg, Germany) with a round section die 1.9 mm × 28 mm was used for filament extrusion. The pre-dried compound material had a moisture content of less than 0.1%. The extrusion temperatures were 200– 190– 190– 190 °C, respectively, from the hopper to the die. A water bath at 70 °C was used in the cooling phase.

The quality was ensured with a standard diameter of the filament using AccuScan 5012 (Beta LaserMike, High Wycombe, UK) for the desired diameter of 1.75 mm ± 0.05 mm. The haul-off unit had a defined force and speed, synchronized with an automated winding unit. Approximately 200 m of each filament (Table 1) was produced. 

In all compositions, 30 wt% of TM wood was used, as this was the percentage that showed the best properties in previous research [30]. 

#### 2.1.4. Fused Filament Fabrication of 3D-Printed Parts

All specimens were modeled in SolidWorks software 2020 (SolidWorks Corp., Waltham, MA, USA) and exported in the STL format. The STL models were sliced and prepared for 3D printing using Cura software version 4.8.0 (Ultimaker, Utrecht, The Netherlands). Specimens were printed using a Creality CR10-V3 3D printer (Creality 3D Technology Co., Ltd., Shenzhen, China) with a direct extruder. The nozzle diameter was 1 mm, the print layer thickness was set to 0.5 mm, the printing temperature to 210 °C, and the bed temperature to 50 °C. The samples were printed with solid layers, with printing lines alternating at an angle of 45° (one layer +45°, next layer −45°) to the specimen length.

#### 2.1.5. Injection Molding

The rheological measurements were carried out on injection-molded test specimens. Extruded filaments were pelletized to a length of 1.5 mm. The discs with a diameter of 25 mm and a thickness of 1.2 mm were produced by melt extrusion in a twin-screw extruder (Xplore Micro compounder MC 15 HT, Xplore Instruments, The Netherlands) in co-rotating operation mode at 180 °C for 5 min at a screw speed of 50 rpm. The homogeneous melt of the sample was transferred to an injection molding piston preheated to 180 °C (Xplore Micro Injection Moulder IM 12, Xplore Instruments, The Netherlands). The sample was formed into a disc geometry with an injection pressure of 900 bar for 10 s and a holding pressure of 1100 bar for a further 10 s. The mold temperature was 60 °C.

### 2.2. Characterization Techniques

#### 2.2.1. Differential Scanning Calorimetry (DSC)

Thermal analysis was performed on wood–PLA and wood–PLA filaments with varying MCC percentages using a Differential Scanning Calorimeter DSC HP1 (Mettler-Toledo, Küsnacht, Switzerland). Around 10 mg specimens were placed in an aluminum crucible performing a thermal cycle under an oxygen atmosphere: heating at 10 °C min^−1^ from 25 to 230 °C; the pressure was not elevated.

The values of glass transition temperature (T_g_), melting temperature (T_m_), and index of crystallinity (X_c_) were determined from the DSC heating curve following Equation (1) [36]:

(1) $$X_{c}=\frac{\Delta H_{m}}{w_{p\;}\times \Delta H_{m}^{0}}\times 100\;\%$$

where w_p_ is the weight fraction of PLA in wood–PLA MCC filament composites, ΔH_m_ is the melting enthalpy of wood–PLA MCC composites, and $\Delta H_{m}^{0}$ the melting enthalpy of 100% crystallized PLA (93.6 J/g).

#### 2.2.2. FT-IR Characterization

Fourier Transform–Infra Red (FT-IR) analysis was used to determine the chemical interactions between the wood particles and PLA–polymer matrix in the composite filaments. The infrared spectra of the wood–PLA composite filament parts were recorded in transmittance mode using a spectrometer Spectrum Two (Perkin Elmer, Waltham, MA, USA). Thirty-two scans were taken at 4 cm^−1^ resolution to capture each spectrum between wavelengths of 4000 and 400 cm^−1^.

#### 2.2.3. Rheology Measurements

All measurements of the rheological properties were performed on rotational controlled rheometer MCR302 (Anton Paar, Graz, Austria). The disc samples were placed directly on a plate–plate sensor system with a diameter of 25 mm (PP25), which was preheated in an inert nitrogen atmosphere to the temperature of the 3D print—210 °C. The rotational flow tests were performed in a shear rate range between 0.001 and 100 s^−1^ at a temperature of 210 °C. The amplitude-dependent oscillation sweep tests were performed in the shear stress range from 0.1 to 1100 Pa (ramp logarithmic), constant frequency 1 Hz, and temperature 210 °C. The dependence of the dynamic storage modulus (*G*′) and loss modulus (*G*″) on frequency was evaluated using frequency tests at a shear stress of 25 Pa, which corresponds to the range of linear viscoelastic response in a frequency range between 0.01 and 100 Hz (ramp logarithmic). The stability of the composites at a temperature of 210 °C was evaluated with the rheological stability test at a constant shear stress of 0.3 Pa and a frequency of 1 Hz over a period of 40 min. All tests were performed to evaluate the effects of MCC on the rheological and mechanical properties of the 3D-printed specimens. 

#### 2.2.4. Flexural Strength and MOE

Flexural strength and modulus of elasticity (MOE) of 3D-printed specimens were tested in a three-point bending test on the Z005 universal testing machine (Zwick-Roell GmbH, Ulm, Germany). Specimens 80 mm × 10 mm × 4 mm in accordance with ISO 178 were used. The support span was 50 mm, and the loading rate was 10 mm/min. The values given for each material were calculated as the average values of 10 specimens. MOE was determined according to EN 310 [37] (Equation (2)):

(2) $$MOE=\frac{L_{s}^{3}\;\left(F_{2}-F_{1}\right)}{4bt\;\left(U_{2}-U_{1}\right)}$$

Ls is the distance between supports [mm];
b and t are the width and thickness of the specimen, respectively [mm];
(F_2_ − F_1_) is the increment of the load on the straight line of the load–deflection curve [N];
(U_2_ − U_1_) is the increment of deflection corresponding to (F_2_ − F_1_) [mm].

#### 2.2.5. Tensile Strength

The tensile tests were performed on 3D-printed specimens (to evaluate the properties of 3D-printed products). Dog bone shapes (type 1BA—75 mm length) complying with the EN ISO 527-2:1996 [38] standard (Plastics—Determination of tensile properties) were used. A test speed of 5 N/min was used. For each material combination, ten 3D-printed specimens were tested using the Z005 universal testing machine (Zwick-Roel GmbH, Ulm, Germany), from which the average values were determined. All tests were carried out at a temperature of 23 ± 2 °C and a humidity of 50 ± 10% on at least 10 specimens in accordance with the EN ISO 527-2:1996 standard.

#### 2.2.6. Scanning Electron Microscopy (SEM)

The microstructure interaction between materials and the influence of MCC on the connection between wood particles and the PLA matrix was visually assessed using a Quanta 250 scanning electron microscope (Thermo Fisher Scientific, Waltham, MA, USA). Filaments and 3D-printed specimens derived from these filaments were cross-sectioned and smoothed using a slide microtome (SM2010R from Leica Microsystems GmbH, Wetzlar, Germany). Prior to SEM observations, the analyzed surfaces were coated with a conductive gold layer. Images of the specimens were captured at various magnifications (75×, 100×, 500×) under high vacuum conditions (1.56 × 10^–2^ Pa) with an electron source voltage of 5.0 kV.

#### 2.2.7. Statistics

Statistical analysis of the data was performed using Microsoft Excel (Microsoft, 2019, Redmond, WA, USA) and PSPP (Gnu Project). The statistical significance of the measured differences was analyzed with basic statistical analysis, and the results were checked with analysis of variance (ANOVA).

## 3. Results and Discussion

### 3.1. DSC Analysis

**Figure 2:**
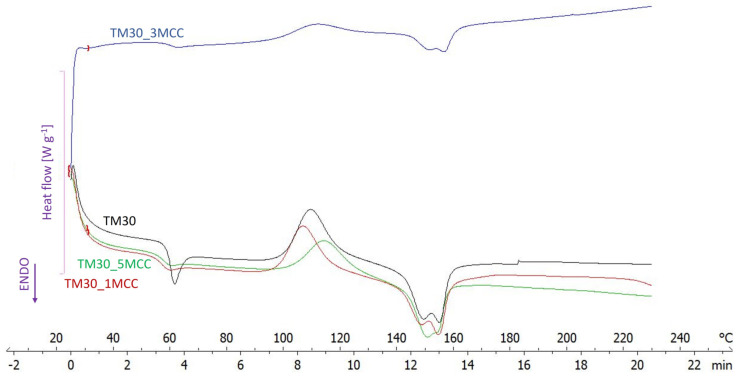
DSC thermograms of TM30 filament, TM30_1MCC filament, TM30_3MCC filament, and TM30_5MCC filament.

PLA, prone to easy crystallization, has the potential to impact the thermal and mechanical characteristics of the composite due to its crystalline nature [39]. In order to investigate the effects of MCC addition on the thermal behaviour of the filaments with different MCC loadings, parts of filaments were subjected to a DSC analysis. The obtained DSC thermograms are presented in Figure 2 and analyzed in Table 2.

With the addition of 1% MCC, the T_g_ decreased (by 1.66 °C) as well as T_m_ (by 1.31 °C). A similar effect was observed when 5% MCC was added, but this was not the case with 3% MCC. After adding 1% MCC to the wood–PLA composite, it formed a crystallization point in the PLA and acted as a nucleating agent, making the PLA crystals more perfect. The chain arrangement was more regular, the crystal size was smaller, and thus the crystallinity improved. However, this trend was not achieved at a higher MCC content, where the MCC particles restricted the movement of the polymer chains and rearranged the crystal lattice structure, resulting in decreased crystallinity. This behavior is in line with a study by Li and co-authors [39]. In a recent study, Ahmad and co-authors [3] found that no chemical changes occurred in the presence of cellulose nanocrystals, but improved crystallinity was observed.

On the other hand, the analysis of the curves by Incarnato and co-authors [25] showed that MCC has no discernible influence on the thermal and crystallization behaviour of PLA, since all materials were completely amorphous, and their transition temperatures (glass transition and melting) were almost the same.

### 3.2. FT-IR Characterization

**Figure 3:**
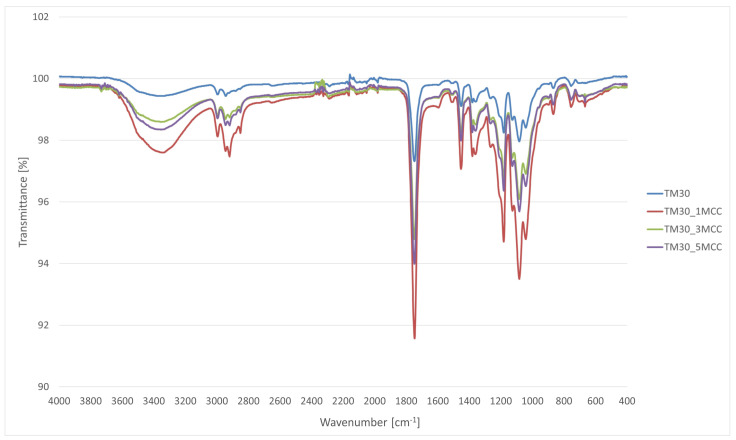
FT-IR spectra of TM30 filament, TM30_1MCC filament, TM30_3MCC filament, and TM30_5MCC filament.

The FT-IR spectra of the wood–PLA filament and wood–PLA filament with varying percentages of MCC are shown in Figure 3. The wood–PLA_MCC filaments showed all the characteristic bands of wood–PLA filament; however, their bands appear to have higher absorption. 

A broad absorption band at 3000–3600 cm^−1^ is observed in the FT-IR spectra, indicating the presence of -OH stretching vibrations of alpha-cellulose when the chemical structure of MCC is analyzed [16].

The first region that appears in all four spectra is the IR band at 1750 cm^−1^, which is due to the characteristic carbonyl (–C=O) stretching bands of PLA. The second region is related to the stretching of the –C–O– bond stretching in the –CH–O– group of PLA at about 1187 cm^−1^, which is associated with the C–O–C stretching. Finally, the last region (common to all four spectra) is associated with the bands at about 1074 cm^−1^ of the C–O–C stretching vibrations. These characteristic bands are also generally found in the literature [40,41,42].

In the TM30_1MCC spectra, the presence of more crystalline order in the MCC samples can be confirmed by the shift of the spectra from 2900 cm^−1^ (C–H stretching vibration of all hydrocarbon constituents in polysaccharides) to a lower wavenumber value [43].

The band associated with a symmetric CH_2_ bending vibration at 1452 cm^−1^ increased in the 1% MCC sample. This band is also known as the crystallinity band [43], where an increase in its intensity indicates a higher degree of crystallinity. Spectra of filament with an addition of 1% MCC therefore show the highest degree of crystallinity. A study from Murphy and Collins [44] confirmed that the addition of 1 and 3 wt% of cellulose causes an increase in crystallinity due to the increased mobility of PLA chains. However, the addition of 5 wt% modified cellulose resulted in restricted chain movement due to PLA/cellulose interactions, leading to a decrease in crystallinity. 

FT-IR bands at least partially overlap, showing the presence of the same functional groups. The FT-IR spectra of TM30 have less peaks than bands associated with the addition of MCC. 

### 3.3. Rheological Characterization

**Figure 4:**
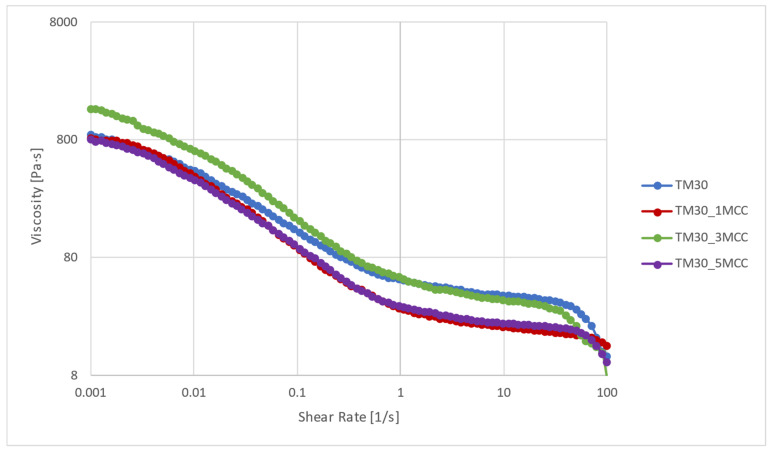
Viscosity curves of TM30, TM30_1MCC, TM30_3MCC, and TM30_5MCC specimens at 210 °C.

Precise viscosity measurements are necessary to control the extrusion of the molten polymer via the print nozzle (with shear rates of 30 to 500 s^−1^) and the entire consolidation process during deposition on the print bed (with shear rates of 0.01 to 0.1 s^−1^) [45,46]. The viscosity of the wood–PLA-based filaments with added MCC is shown in Figure 4. The viscosity of all samples decreased by about one and a half decades with an increasing shear rate, which is typical for shear thinning behaviour. The viscosity of the composite samples decreased as a result of the reduction in polymer chain mobility at the interface of the components [47]. The viscosity at a shear rate of 0.001 s^−1^ was about 800 Pa·s for TM30, TM30_1MCC, and TM30_5MCC, while it was slightly higher for TM30_3MCC (1500 Pa·s). The addition of 1 and 5% MCC did not significantly change the viscosity at lower shear rates, but at a shear rate higher than 0.1 s^−1,^ the viscosity decreased compared to the sample without MCC (TM30). However, the addition of 3% MCC increased the viscosity at lower shear rates, while at higher shear rates, no effect of the MCC addition on the viscosity can be seen. The viscosity results show that the composites exhibit non-Newtonian behavior, and the addition of MCC does not significantly improve the flow resistance. In the composites with 1 and 5% MCC, the polymer chains and the MCC formed a special structure (spherulite) around the TM particles, which was distorted at a higher shear rate due to the increasing disentanglement [24].

With increasing shear rate, viscosity decreased in all blends, which can be attributed to reduced chain mobility at the interface influenced by refined segmentation of PLA by TM wood particles. This effect is consistent with previous findings [24,47]. The MCC-loaded biocomposites exhibited liquid-like behavior, which can be attributed to the lower mobility of the polymer chains. The different sizes of the MCC and TM wood particles, together with the molten polymer, formed spherical structures that enhanced distortion and disentanglement. At higher shear rates, the complex viscosity decreased as the polymer chains moved more independently within these structures. There are several possible reasons for the decrease at the end of the curve; wood particles can cause filler–filler and filler–polymer interactions that significantly affect the final properties. Filler–filler interactions lead to clustering, which requires high stress to break it and allow flow. Filler–polymer interactions increase the internal bonds, making it harder to re-form once broken, resulting in higher viscosity and dynamic moduli. This leads to a Newtonian-like plateau in the viscosity curve that ends at a lower frequency, as discussed in [48].

**Figure 5:**
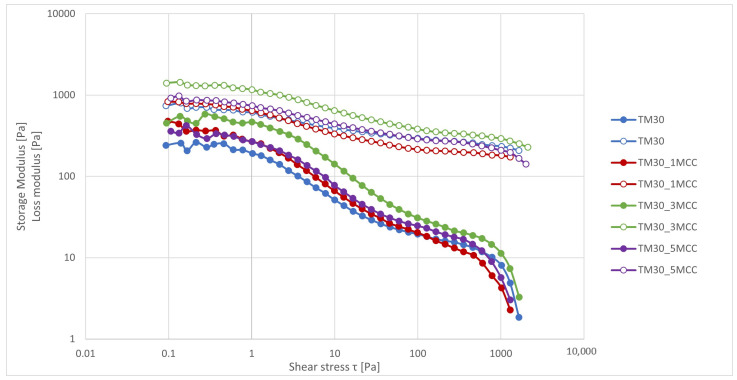
Amplitude tests. Storage (filled circles) and loss (empty circles) modulus dependent on shear stress for TM30, TM30_1MCC, TM30_3MCC, and TM30_5MCC specimens at 210 °C.

The dynamic viscoelastic modulus, i.e., the storage and loss modulus in the shear stress range between 0.1 and 1000 Pa, is shown in Figure 5. The values of the storage modulus, which describes the capacity to accumulate the elastic part of the energy, have a lower value over the entire measurement range than the loss modulus, which describes the capacity to dissipate the viscous part of the energy. With the addition of 3% MCC, a higher value of the storage modulus was observed. This means that the strongest interaction of MCC, and thus the highest rigidity of the polymer chains, was present. The loss modulus of the composites also decreased with increasing shear stress. The addition of 1% MCC decreased the loss modulus, while the addition of 3% MCC increased the loss modulus throughout the shear stress range. After the addition of 3% MCC to the composite, circular shape structures (agglomerates) were formed, which also inhibited the movement of the polymer chains. This observation is also consistent with the shift in T_g_ temperature from 61.81 °C to 63.36 °C for TM30 (without MCC) and TM30_3MCC, indicating that minimal mobility of the polymer chains and lower crystallinity was observed at this MCC concentration. The addition of 1% MCC lowered the T_g_ temperature and increased the crystallinity of the composite, indicating the best polymer chain mobility and crystal lattice structure. In earlier research, it was reported that the G′ value of pure PLA was 0.83 GPa [49] or 1.04 GPa [30].

**Figure 6:**
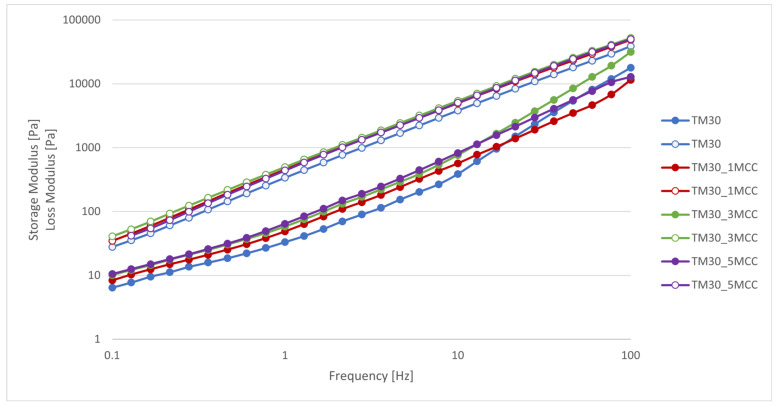
Frequency sweep tests. Storage (filled circles) and loss (empty circles) modulus as a function of frequency at a temperature of 210 °C for TM30, TM30_1MCC, TM30_3MCC, and TM30_5MCC samples.

The storage and loss moduli as a function of frequency in a range between 0.1 and 100 Hz at a constant shear stress of 25 Pa are shown in Figure 6. The loss modulus is dominant over the storage modulus in the entire frequency range, which is typical for the liquid-like behavior of composites at the 3D printing temperature of 210 °C. The results of the frequency tests show that the addition of MCC increases the ability to store energy, which means that the MCC stabilizes the structure of the composites.

In a higher frequency range, the storage modulus of the TM30_3MCC composite was the highest, which is due to the higher degree of molecular entanglement of the short polymeric chains inside the circular agglomerates. At frequencies below 10 Hz, the stability of the composite samples increased slightly due to the higher MCC content, as the storage modulus increased proportionally to the higher MCC content. The loss modulus of the composites increased with increasing frequency as the viscosity of the composites decreased. With a higher segment mobility, which was limited by a higher MCC content, the mechanical behavior, i.e., the flexural and tensile strength, also improved.

**Figure 7:**
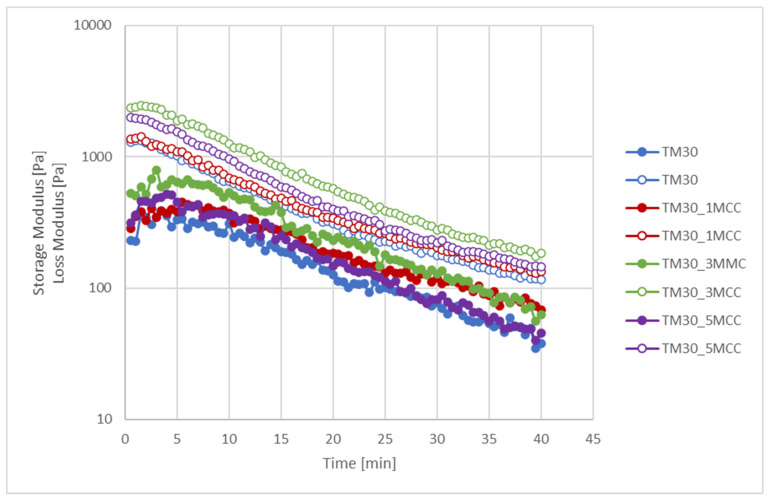
Stability tests. Storage (filled circles) and loss (empty circles) modulus as a function of time at a constant temperature of 210 °C for TM30, TM30_1MCC, TM30_3MCC, and TM30_5MCC samples.

The results of the stability test, i.e., the storage and loss modulus as a function of time at non-destructive conditions (frequency 1 Hz and shear stress 0.3 Pa), are shown in Figure 7. The observed unstable behaviour of the composites is due to the difference in the density of the components. The lower density was obtained in the MCC (~150–220 kg/m^3^), which reached the fast sedimentation in the polymer matrix PLA, with a 10-times higher density (1240 kg/m^3^). Of the composites, 30% consisted of thermally modified wood with a density of about 600 kg/m^3^. After the first 5 min, the decrease of both moduli was the result of the sedimentation of MCC and TM in the polymer matrix. However, these long times are not relevant for 3D printing and are not important for successful printing.

### 3.4. Flexural Strength and MOE Results

**Figure 8:**
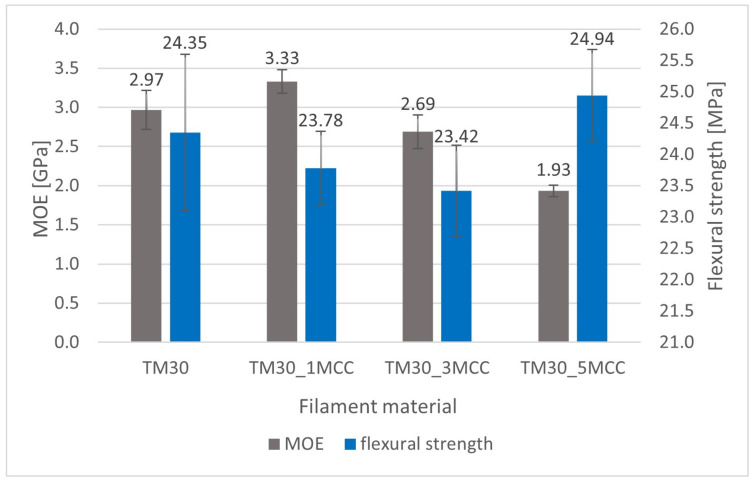
MOE (left) and flexural strength (right) of specimens from wood–PLA filaments and wood–PLA with 1%, 3%, and 5% of MCC content.

The MOE increased with the addition of 1% MCC and decreased with a higher content of MCC (Figure 8). The highest MOE was obtained for the specimens TM30_1MCC (3.33 GPa), i.e., 12.1% higher than reference TM30. However, differences among all the investigated specimens were significant (ANOVA, *p* = 0.000). Furthermore, also in Fisher’s LSD, the differences between all investigated specimens were statistically significant (ANOVA, *p* ≤ 0.004)

Flexural strength is 2.42% higher for specimens 3D-printed from filament with 5% MCC additive than specimens from wood–PLA without MCC. This is in line with our expectations, as we assumed that MCC acts as a natural compatibilizer with a crosslinking function. Nevertheless, specimens from filaments with lower content of MCC (1 and 3%) had 2.34% and 3.82% lower flexural strength, respectively. The differences among the investigated specimens were significant (ANOVA, *p* = 0.003). In Fisher’s LSD, the differences between TM30 and TM30_3MCC were statistically significant (ANOVA, *p* = 0.024), also between TM30_1MCC and TM30_1MCC (ANOVA, *p* = 0.007) and finally between TM30_3MCC and TM30_5MCC (ANOVA, *p* = 0.000).

### 3.5. Tensile Strength Results

Cellulose, the main component of natural fibers, has a strength and stiffness of >2 GPa and 138 GPa, respectively [50]. However, the stiffness of these natural fibers mainly depends on the microfibril angle, so fibers with high cellulose content and low microfibril angle have a high reinforcing effect in polymer composites.

**Figure 9:**
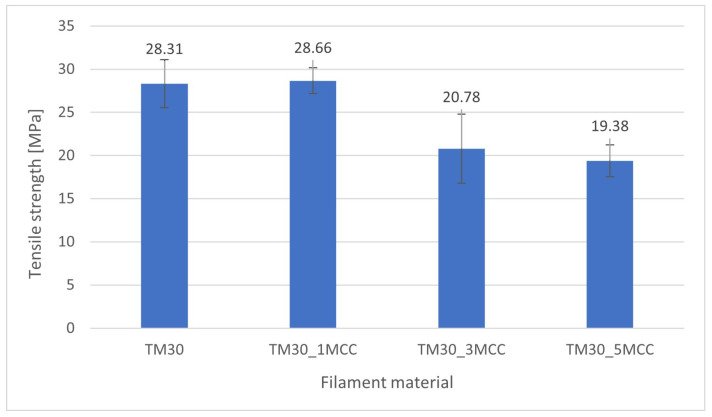
Tensile strength of specimens from wood–PLA filaments and wood–PLA with 1% of MCC, 3% of MCC, and 5% of MCC content.

The tensile strength (Figure 9) slightly increased (for 1.24%) in specimens with 1% MCC. In specimens with 3% and 5% of MCC, tensile strength was reduced by 26.6% and 31.5%, respectively. Nevertheless, it should be noted that with TM30_3MCC and TM30_5MCC, not only the higher addition of MCC but also the lower weight proportion of PLA has an influence on the specimens’ strength (see Table 1). However, differences among the investigated specimens were significant (ANOVA, *p* = 0.000). In Tukey’s HSD, the differences between TM30 and TM30_1MCC were not statistically significant (ANOVA, *p* = 0.993), and between TM30_3MCC and TM30_5MCC were also statistically different (ANOVA, *p* = 0.718).

A recent study by Ahmad and co-authors [3] revealed that the tensile strength of the neat PLA filament was increased by 18.2% with the addition of up to 1 wt% cellulose nanocrystals (CNCs) but decreased at a content of 2 wt% CNCs. In the 3D-printed samples, the tensile strength of the pure PLA filament was improved by 11% with the introduction of 0.75 wt% CNCs and decreased with further CNC additions.

### 3.6. Scanning Electron Microscopy (SEM) Analysis

**Figure 10:**
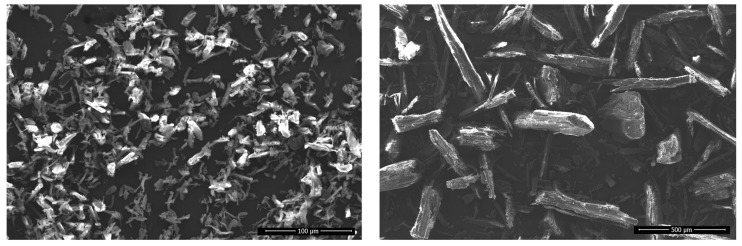
Scanning electron micrographs of MCC (**left**) and TM wood particles (**right**).

Electron micrographs of MCC (type UFC 100) and of TM wood particles are shown in Figure 10. It is evident that both the MCC and TM wood particles are elongated; their aspect ratio—the ratio of length to width—is quite high. This indicates their reinforcement potential.

**Figure 11:**
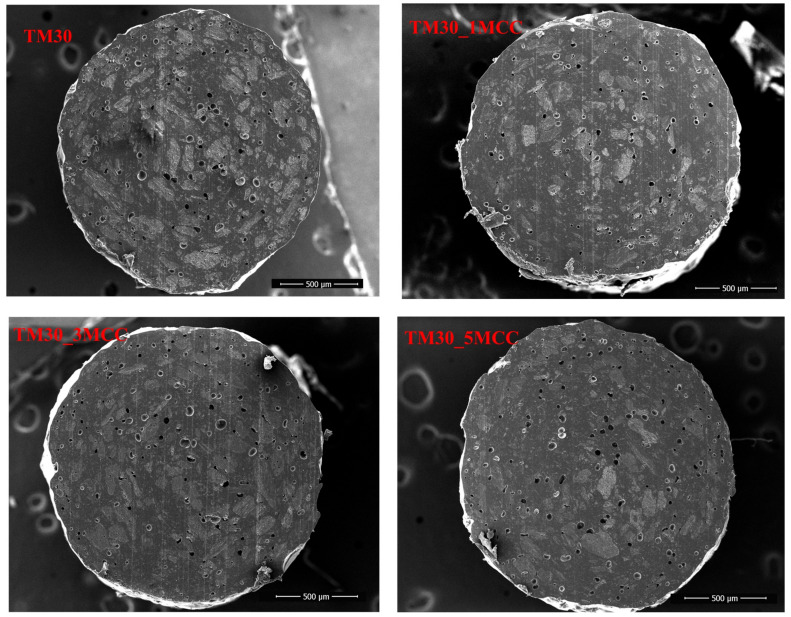
Cross-section of filament from wood–PLA (TM30) and wood–PLA filaments with added MCC (TM30_1MCC, TM30_3MCC, and TM30_5MCC).

The cross-section of four filaments and 3D-printed specimens from these filaments were studied with SEM. No meaningful differences were found in the electron micrographs of filaments under low magnification (Figure 11). However, there is a noticeable contrast in the size and quantity of voids, which appear to be the fewest and smallest within the cross-section of the TM30_1MCC filament.

**Figure 12:**
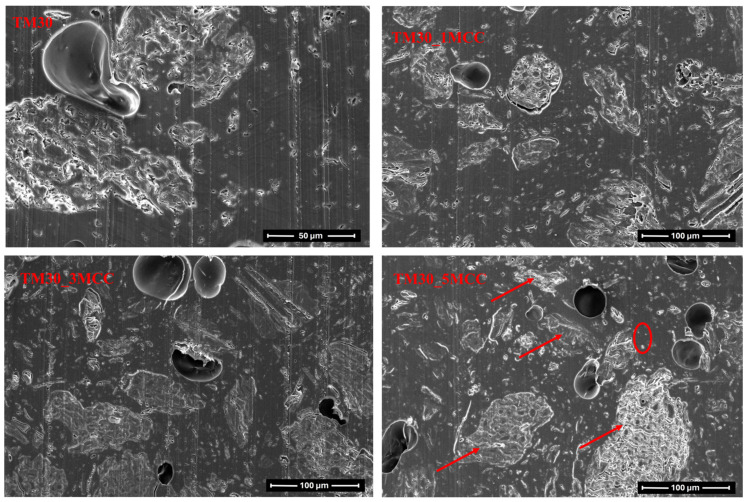
Cross-section of filament from wood–PLA (TM30) and wood–PLA filaments with added MCC (TM30_1MCC, TM30_3MCC, and TM30_5MCC); 500× magnification. An oval encloses an area with different MCC particle sizes and arrows pointing at TM wood particles.

Microscopic observation showed that the MCC is well distributed and dispersed in the PLA matrix with 30% TM wood particles (Figure 12). It does not concentrate around the wood particles but is agglomerated in some cases, especially at higher MCC contents, and therefore probably cannot serve as reinforcement of the matrix. A study by Fortunati and co-authors [51] found that when the small particles agglomerate into larger particles, they can no longer act as nucleating agents.

Cellulose serves as a mechanical reinforcement in polymer composites, leading to a considerable improvement in the composite’s tensile modulus [52,53,54]. The mechanical properties of polymer composites are influenced by factors such as filler dispersion and the size of cellulose fiber particles within the polymer matrix. It is well established that for cellulose micro- and nanoparticles to effectively reinforce a composite, they must be uniformly dispersed in the polymer matrix. This uniform dispersion is essential as it prevents the creation of substantial stress concentrations in the polymer matrix. Consequently, well-dispersed cellulose particles, due to their small size, can positively impact tensile properties without introducing stress concentration points [55]. Mechanical properties improvements rely on careful control of cellulose particle dispersion during the composite manufacturing process, utilizing appropriate processing techniques and additives to ensure uniform distribution and prevent agglomeration in the polymer matrix. 

However, the presence of voids is also evident. There are probably two reasons for the formation of voids. Firstly, the voids were formed when the melted filament was extruded from the nozzle. The voids then increased in size as the melt left the die, as the pressure difference between the inside and outside of the die allowed the voids to form. Secondly, void formation was also attributed to the poor interfacial adhesion between reinforcing materials and matrix in the composite systems [3].

**Figure 13:**
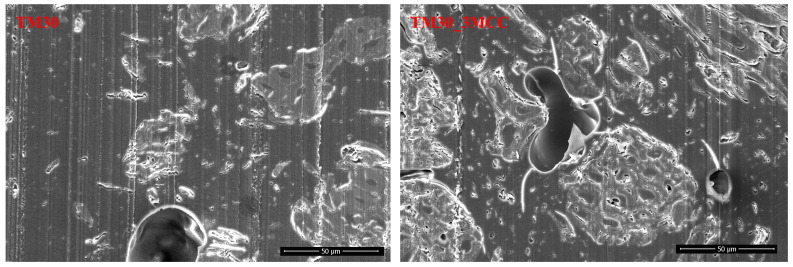
Cross-section of filament from wood–PLA (TM30) and wood–PLA filament with 3% MCC addition (TM30_3MCC), 1000× magnification.

With 1000× magnification (Figure 13, right), despite void formation, there seems to be quite good bonding between PLA and MCC particles. Ahmad and co-authors [3] reported a weak bonding between the PLA matrix and the MCC particles due to the polarity difference, where PLA was a non-polar polymer, and MCC was polar due to the hydroxyl groups on its surface. This resulted in agglomeration of the MCC particles and voids or gaps between the interface of the PLA matrix and the MCC particles. The presence of voids further affected the mechanical properties of the extruded filaments.

**Figure 14:**
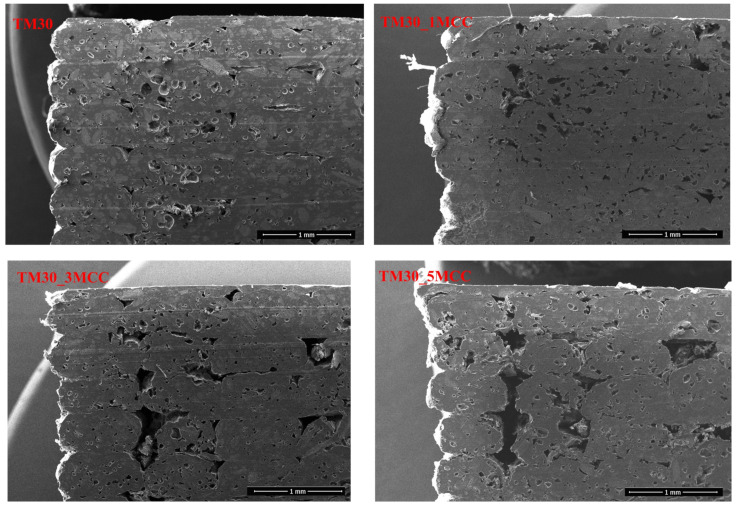
Cross-section of 3D-printed rectangular specimens from wood–PLA (TM30) filament and wood–PLA filaments with added MCC (TM30_1MCC, TM30_3MCC, and TM30_5MCC).

The microscopic images of the 3D-printed specimens (Figure 14) show that the specimens made of filaments with a higher MCC content (TM30_3MCC and TM30_5MCC) exhibited larger voids and delamination of the printing layers. The porosity of the 3D-printed parts is larger than the porosity of filaments. Part of the porosity is due to pre-existing voids in the filaments, as there is no degassing in the printer’s extrusion nozzle, and air pockets from the filaments are “transferred” to the 3D-printed part, part from possible moisture in the filaments and the formation of gasses during printing, part from possible uneven flow and partial clogging of the nozzle by wood particles and also from raster gap voids [56] These observations are consistent with most of the earlier results of this study, as this is also a reason for lower mechanical properties.

## 4. Conclusions

FT-IR spectra of the filament with an addition of 1% MCC exhibit the highest intensity of peaks associated with crystallinity and therefore show the highest degree of crystallinity. This was also confirmed by DSC thermograms, which additionally showed its lower glass transition temperature (by 1.66 °C) and lower melting temperature (by 1.31 °C). The addition of 1% MCC leads to an increase in crystallinity due to increased mobility of the PLA chains. Three-dimensionally printed specimens from this filament also yielded the highest tensile strength (1.2%) and MOE (12.1%). However, the addition of 3 and 5% MCC resulted in restricted chain movement due to PLA/MCC interactions—the mobility of the polymer chains of the matrix was inhibited by hydrophilic MCC—leading to a decrease in crystallinity and subsequently lower mechanical properties, i.e., tensile strength, and MOE. This is also in line with rheological analysis, which showed that the highest value of the storage modulus was noted with the addition of 3% MCC. It indicates the presence of the strongest MCC interaction and, consequently, the highest level of chain rigidity. However, the loss modulus was reduced by the addition of 1% MCC, indicating the best mobility of the polymer chains and its best crystal lattice structure. The results of the frequency tests show that the addition of MCC increases the ability to store energy, which means the MCC stabilizes the structure of the composites. Based on the viscosity results, it can be concluded that the composites exhibit non-Newtonian characteristics. Furthermore, the inclusion of MCC does not appear to have a substantial effect on the flow resistance. According to the SEM images, the MCC particles are quite well dispersed in the polymer matrix, with some agglomeration occurring, which does not affect the properties of the composite at low MCC loading.

A next step in our future research should include the use of surface-treated cellulose, for example, acetylated freeze-dried nanofibrillated cellulose. 

## Figures and Tables

**Table 1:** Formulations and material compositions of prepared filaments.

Formulation Code	Material Compositions (wt%)		
	PLA	TM Beech	MCC
TM30	70	30	-
TM30_1MCC	69	30	1
TM30_3MCC	67	30	3
TM30_5MCC	65	30	5

**Table 2:** Glass transition temperature (T_g_), melting temperature (T_m_), melting enthalpy (ΔH_m_), and index of crystallinity (X_c_) for TM30 filament, TM30_1MCC filament, TM30_3MCC filament, and TM30_5MCC filament.

Filament	T_g_ [°C]	T_m_ [°C]	ΔH_m_ [J/g]	X_c_ [%]
TM30	61.81	155.52	8.34	12.73
TM30_1MCC	60.15	154.21	11.47	17.76
TM30_3MCC	63.36	156.78	3.37	5.37
TM30_5MCC	60.67	150.57	7.62	12.52

## Data Availability

Data are contained within the article.
